# Genetic Variations of *TAP1* Gene Exon 3 Affects Gene Expression and *Escherichia coli* F18 Resistance in Piglets

**DOI:** 10.3390/ijms150611161

**Published:** 2014-06-20

**Authors:** Qiaohui Zhao, Ying Liu, Wenhua Dong, Shiping Zhu, Yongjiu Huo, Shenglong Wu, Wenbin Bao

**Affiliations:** Key Laboratory for Animal Genetics, Breeding, Reproduction and Molecular Design, College of Animal Science and Technology, Yangzhou University, Yangzhou 225009, China; E-Mails: zqh200848@163.com (Q.Z.); yddkly@163.com (Y.L.); dingdang626@163.com (W.D.); shiping_zhu@163.com (S.Z.); yjhuo@yzu.edu.cn (Y.H.)

**Keywords:** piglets, *TAP1* gene, *E. coli* F18, genetic variation, mRNA expression

## Abstract

Firstly, our research group identified Sutai pigs’ phenotypes that exhibited extreme resistance and susceptibility to the *Escherichia coli* F18 respectively, and then eight *ETEC* (*Enterotoxigenic Escherichia coli*) F18-resistant piglets and eight *ETEC* F18-sensitive piglets were selected. Then, the *TAP1* (*Transporter associated with antigen processing*) mRNA relative expression levels were analyzed in 11 tissues of the resistant and susceptible phenotypes. Simultaneously, we detected the genetic variations in exon 3 of the *TAP1* gene and evaluated the *TAP1* mRNA expression levels among the different genotype pigs to study the effects of the genetic variation on gene expression, and the *E. coli* F18 resistance. The results revealed higher expression levels in the resistant genotypes than that in the susceptible genotypes in 11 tissues, with significant differences in the spleen, lymph node, lung, thymus, duodenum and jejunum*.* Furthermore, a G729A mutation was identified in the *TAP1* gene exon 3, and this mutation deviates from Hardy-Weinberg equilibrium (*p* < 0.01). The *TAP1* mRNA levels in GG genotype were significantly higher than that in the other two genotypes, with significant differences in the liver, lung, kidney, thymus, lymph node, duodenum and jejunum tissues. We speculated that high expression of the *TAP1* gene might confer resistance against the *E. coli* F18, the G729A mutation had a significant effect on the mRNA expression, and individuals with the GG genotype possessed a stronger ability to resist the *E. coli* F18 infection.

## 1. Introduction

Transporter associated with antigen processing (TAP) is a protein transporter that consists of two protein subunits, TAP1 and TAP2. Both these subunits have an *N*-terminal membrane-spanning domain and a *C*-terminal nucleotide-binding domain, and together they form a heterodimer that plays a pivotal role in intracellular antigen presentation by translocating intracellular antigenic peptides from the cytosol into the lumen of the endoplasmic reticulum (ER) [1]. TAP1 is responsible for the transport of peptides generated by proteasomal degradation to the ER lumen, after which they are transported to the cell surface in conjunction with MHC class I molecules, inducing specific recognition and processing by CD8^+^ T cells and NK cells, after which the target cell is lysed and the virus is killed [2]. MHC class I molecules require TAP1 to overcome their structural instability during maturation and transportation. Previous studies on humans have indicated that mutations in the *TAP1* gene affect antigen peptide selection and the transport of antigenic peptides into the ER, leading to susceptibility to disease [3,4]. Vaske *et al*. reported the *TAP1* gene polymorphisms in Yorkshire, Duroc and other Western pig breeds [5]. Furthermore, the *TAP1* gene has been studied as one effective functional gene for disease resistance in pigs [6,7]. Wysocki [8] *et al.* found the *TAP1* gene up-regulated in lung tissue when pig infected by porcine reproductive and respiratory syndrome virus (PRRSV). Uthe *et al.* [9] found the *TAP1* gene might play an important role in *Salmonella* resistance. Wang [10] *et al.* analyzed dynamic changes in *TAP1* expression levels in newborn to weaning piglets, and its association with *Escherichia coli* F18 resistance. The Sutai pig, which was generated by crossing Duroc pigs with Taihu pigs, is a high-quality and lean-meat breed. Through five years of continuous breeding, *E. coli*-resistant and *E. coli*-susceptible resource groups have been created in a population of more than 200 Sutai pigs [11]. At the same time, we also constructed a V-secretion system based on the expression of *E. coli* F18 adhesin; using the V-secretion system and the receptor binding assay, further analysis and verification of *E. coli* F18 resistance and susceptibility were performed for these resource groups [11,12]. In this study, we analyzed the *TAP1* gene relative expression levels in 11 tissues among extreme phenotypes (resistance and susceptibility to the *Escherichia coli* F18) individuals. Simultaneously, we detected genetic variations in exon 3 of the *TAP1* gene in Sutai pig breed and analyzed the *TAP1* gene mRNA relative expression levels among Sutai pigs of different genotypes to provide a theoretical and experimental basis of the applicability of the *TAP1* gene as a genetic marker in breeding practices and future functional analysis.

## 2. Results

### 2.1. Identification of the Escherichia coli F18-Resistant and E. coli F18-Susceptible Sutai Piglets

**Figure 1:**
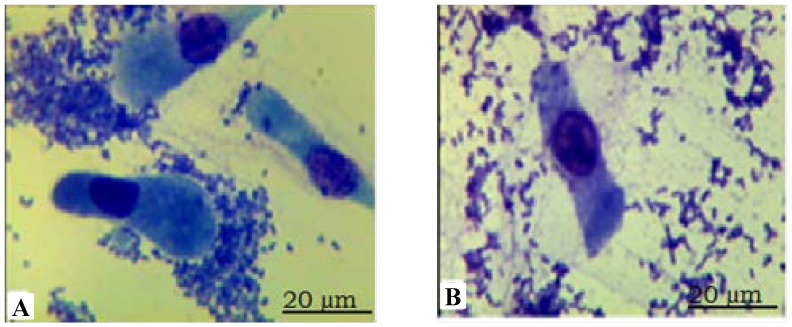
The adhesion of *Escherichia coli* F18 to intestinal epithelial cells in Sutai piglets, **A** represents susceptible piglets; **B** represents resistant piglets. Photos were taken with an oil immersion lens at 1000× magnification, and the scale bar was 20 μm.

The adherence assay showed that small intestinal epithelial cells from eight *ETEC* F18-resistant piglets in this experiment displayed nearly no adherence with *E.coli* bacteria. In contrast, intestinal epithelial cells from eight *ETEC* F18-sensitive piglets showed clear adherence with F18ab-expressing fimbriae of the standard *ETEC* strain (Figure 1).

**Table 1:** Analysis of the *TAP1* gene mRNA expression levels in 11 tissues of the *E. coli* F18-resistant and *E. coli* F18-susceptible.

Tissues	Identified Groups (Number)		Difference Multiples
	Resistant (8)	Susceptible (8)	
Heart	2.49 ± 0.94	1.74 ± 0.81	1.43
Liver	5.98 ± 1.65	4.75 ± 1.37	1.25
Spleen	25.86 ± 6.52 ^a^	13.21 ± 5.28 ^b^	1.96
Lung	34.65 ± 8.24 ^a^	18.28 ± 6.64 ^b^	1.90
Kidney	12.14 ± 2.51	9.19 ± 2.01	1.32
Stomach	9.85 ± 2.32	7.21 ± 2.74	1.37
Muscle	1.21 ± 0.45	1.00 ± 0.00	1.21
Lymph	36.85 ± 7.55 ^a^	16.44 ± 4.38 ^b^	2.24
Thymus	27.21 ± 4.21 ^a^	11.52 ± 3.21 ^b^	2.37
Duodenum	28.52 ± 9.88 ^a^	15.48 ± 6.58 ^b^	1.84
Jejunum	32.19 ± 4.58 ^a^	16.59 ± 7.05 ^b^	1.94

values in the same row with different superscript letters mean significant difference (^a^ *p* < 0.05 compared with the susceptible group; ^b^ *p* < 0.05 compared with the resistant group).

### 2.2. TAP1 Gene mRNA Expression Levels in 11 Tissues of the E. coli F18-Resistant and E. coli F18-Susceptible Sutai Piglets

Real-time PCR was used to detect the mRNA relative expression of the *TAP1* gene in 11 tissues of Sutai pigs. The mRNA relative expression of the *TAP1* gene in muscle tissue was used as a reference. As shown in Table 1, in both *E. coli* F18-resistant and *E. coli* F18-susceptible individuals, the *TAP1* gene mRNA relative expression levels in the small intestine (duodenum and jejunum), immune tissues (spleen, thymus and lymph nodes), and lungs were high. The mRNA relative expression levels of the resistant piglets were higher than that of the susceptible piglets, with difference multiples ranging from 1.21 to 2.37. Furthermore, the mRNA relative expression levels in the spleen, lymph node, lung, thymus, jejunum and duodenum significantly differed between the resistant and susceptible genotypes (*p* < 0.05).

### 2.3. Analysis of PCR-RFLP and Sequencing Results

**Figure 2:**
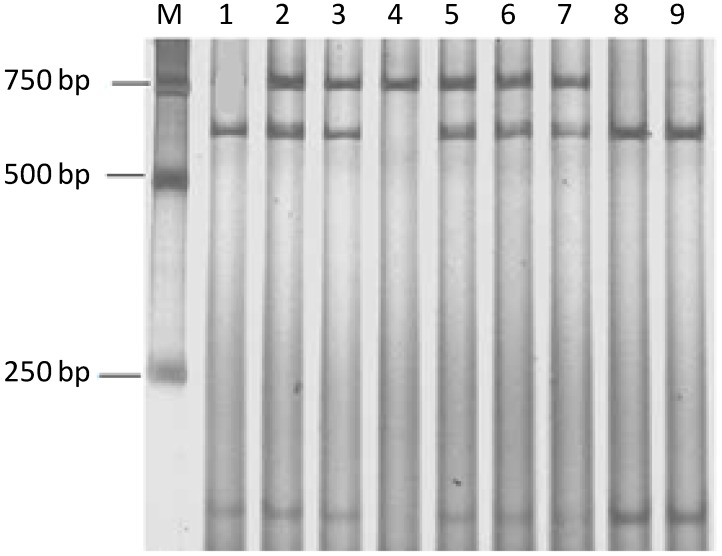
Polymerase chain reaction-restriction fragment length polymorphism (PCR-RFLP) results of the *TAP1* gene G729A locus AA-type (767 bp) is in lane 4; GG-type (628 bp/139 bp) are lane 1, 8, 9; and AG-type (767 bp/628 bp/139 bp) are in lanes 2, 3, 5, 6, 7. M represents pBR322 DNA/*Bsu*R (*Hae*III) Marker.

PCR amplification was carried out using the designed primers. The length of the amplified fragment obtained by electrophoresis was in agreement with the predicted fragment length 767 bp, and there were no nonspecific bands. Based on the PCR products, polymerase chain reaction-restriction fragment length polymorphism (PCR-RFLP) analysis was performed and three genotypes were found among the Sutai piglets: GG (628/139 bp), AA (767 bp), and AG (767/628/139 bp). The results of the PCR-RFLP are shown in Figure 2.

The PCR products from the AA and GG homozygotes were sent to Sangon Biotech (Shanghai, China) for sequencing on an ABI PRISM 377 automatic DNA sequencer. According to the identical sequence from GenBank (BX323833), there exists one A/G substitution mutation at nucleotide 729 (start from gene initiator codon: ATG), and the SNP G729A is a synonymous mutation.

### 2.4. Analysis of Gene Frequency Distribution and Hardy-Weinberg Equilibrium of the TAP1 Gene Exon 3 G729A Mutation

As shown in Table 2, three genotypes were present in the Sutai pigs, the statistical analysis for PCR-RFLP genotypes and their frequencies in Sutai pigs *TAP1* gene indicated that the frequencies of AA, AG, and GG genotypes were 0.603, 0.275 and 0.122 respectively. Allele A is the dominant allele. According to the χ^2^ goodness-of-fit test, the group of Sutai pigs showed a significant deviation from Hardy-Weinberg equilibrium (*p <* 0.01).

**Table 2:** Genetic variation analysis of the *TAP1* gene exon 3 G729A mutation in Sutai pigs.

Sample Number	Genotype Frequency			Allele Frequency		χ^2^ Value
	AA	AG	GG	A	G	
196	0.602 (118)	0.276 (54)	0.122 (24)	0.740	0.260	15.851

*χ* ^2^_0.05_ (1) = 3.84; *χ* ^2^_0.01_ (1) = 6.63.

### 2.5. Levels of TAP1 mRNA Relative Expression in Different Genotypes

The *TAP1* mRNA relative expression levels in the spleen, thymus, lung, lymph node, duodenum, and jejunum were high among all the different genotypes. As shown in Table 3, the mRNA relative expression levels of the *TAP1* gene were higher in the GG genotype and AG genotype than that in the AA genotype in the heart, and the relative expression levels in the GG genotype were significantly higher than those in the AA and AG genotypes in the liver, lung, kidney, thymus, lymph node, duodenum, and jejunum tissues (*p* < 0.05).

**Table 3:** Differential *TAP1* mRNA expression levels in 11 tissues among different genotypes in Sutai pigs.

Tissues	Genotype (Number)		
	AA (8)	AG (8)	GG (8)
Heart	0.69 ± 0.09 ^a^	3.19 ± 1.73 ^b^	3.40 ± 2.01 ^b^
Liver	3.21 ± 1.45 ^a^	7.76 ± 5.49 ^a^	19.38 ± 12.32 ^b^
Spleen	10.91 ± 0.30	23.82 ± 13.68	65.28 ± 23.47
Lung	26.77 ± 7.40 ^a^	25.37 ± 5.94 ^a^	101.39 ± 41.12 ^b^
Kidney	6.75 ± 0.82 ^a^	11.46 ± 5.98 ^a^	25.81 ± 23.31 ^b^
Stomach	8.27 ± 1.39	21.13 ± 12.29	51.87 ± 20.91
Muscle	1.00 ± 0.00	1.07 ± 0.23	1.03 ± 0.15
Thymus	17.54 ± 6.00 ^a^	18.74 ± 7.59 ^a^	61.16 ± 19.34 ^b^
Lymph node	31.18 ± 21.72 ^a^	25.39 ± 15.01 ^a^	75.15 ± 21.3.8 ^b^
Duodenum	43.14 ± 4.68 ^a^	30.22 ± 12.18 ^a^	59.97 ± 15.26 ^b^
Jejunum	30.16 ± 0.28 ^a^	31.14 ± 15.51 ^a^	89.91 ± 15.07 ^b^

Values in the same row with different superscript letters indicate significant difference (^a^ *p* < 0.05 compared with the AG/GG genotype groups; ^b^ *p* < 0.05 compared with the AA/AG genotype groups).

## 3. Discussion

The real-time PCR results revealed high mRNA relative expression levels of the *TAP1* gene in the small intestine (duodenum and jejunum), immune system (spleen, thymus, and lymph nodes), and lung tissues. The immune system tissues were the main sites of resistance, and immune cells generate many immune factors that are then distributed to various organs for immune function of the body. The liver and kidney are the most important detoxification organs, metabolic wastes and harmful substances are excreted from the body from these organs. The duodenum and jejunum exist in a complex environment with a large number of diverse microorganisms. Therefore, it is necessary for the small intestine to maintain normal immune metabolism under complex environment. As a vital organ in the respiratory system, lungs frequently communicate with the outside environment and neutrophils and T cells could constantly produce high levels of immune factors in response to various external stimuli; nevertheless, this does not occur in lungs [13]. Sun *et al.* used real-time PCR and reported high *TAP1* gene mRNA relative expression levels in the immune system and lung tissues in crossbred Chinese pigs [6]. Furthermore, Du *et al.* found a close association between the pulmonary immune and lymphoid immune channels in enterogenous infection, and intestinal tissues are also known as “pro-inflammatory organs” [14]. Therefore, as *TAP1* appears to play an important role in the endogenous and exogenous antigen presentation processes, it follows that it would be expressed at high levels in lung, immune system tissues, and intestinal tissues.

Previous researches have demonstrated that low expression levels of TAP can result in the viruses or cancer cells being ignored by the immune system [15,16,17]. Sun *et al.* found that lipopolysaccharide (LPS) secretion resulted in increased expression of the *TAP1* gene mRNA in porcine kidney cells (PK-15) [6]. In addition, researchers have reported that *TAP1* gene mRNA expression increased significantly in porcine pulmonary cells infected by *Salmonella* and influenza H1N1 virus [18,19]. Schultz and Salle *et al.* found that the function of the BPI protein was inhibited among TAP-deficient patients, who were more vulnerable to infections of gram-negative bacteria [20,21,22]. All these studies suggested that up-regulation of *TAP1* gene expression played an important role in immune defense prevent bacterial and viral infections. In our study, the mRNA expression levels were higher in the resistant genotype than the susceptible genotype, and the difference multiples among the 11 tissues examined ranged from 1.21 to 2.37. Meanwhile, there were significant differences in the spleen, lymph node, lung, thymus, jejunum, and duodenum between the resistant and susceptible genotypes (*p <* 0.05). These results indicated that the higher mRNA expression level might confer resistance to pathogenic (including the *E. coli* F18) infection.

Simultaneously, the mRNA expression levels of the *TAP1* gene were higher in the GG genotype than that in the AA and AG genotypes, and existed significant difference in the liver, lung, kidney, thymus, lymph node, duodenum, and jejunum tissues (*p <* 0.05). Although the mutation, which is a synonymous mutation, was in exon 3 of the *TAP1* gene, such mutations do not result in an altered amino acid; However, such mutations in the coding region might affect the translation efficiency of the same amino acid or the transcription level of the gene, thereby causing changes in the protein expression level, consequently affecting the normal physiological functions of the body [23]. Therefore, the G729A mutation might upregulate the *TAP1* mRNA expression in various tissues, resulting in the high mRNA expression level in the liver, lung, kidney, and immune tissues (thymus and lymph node) of the GG genotype piglets, which may play an important role in heightening the ability of resistance to pathogenic infection. The *E. coli* F18 is a one of the main causative agents of diarrhea and edema. Specifically, fimbriated *E. coli* F18 can adhere to enterocyte receptors, and subsequently reproduce and produce enterotoxins that cause disease [24]. Intestinal epithelial cells activate the body’s adaptive immunity through recognition and antigen presentation of the *E. coli* enterotoxin. The activation of nuclear factor kappa B (NF-κB) then triggers the expression of cytokines such as IL-6, TNF-α, and IFN-γ are then synthesized and released outside the cell. These cytokines enhance the chemotactic aggregation of granulocytes and macrophages, capillary permeability, and lymphocyte infiltration in order to resist *E. coli* F18 infection. The TAP1 molecules play a pivotal role in antigen presentation, which has been widely demonstrated in human medicine. Therefore, the *TAP1* gene exon 3 G729A polymorphism might confer stronger resistance of GG genotype piglets to the *E. coli* F18.

Gene frequency distribution analysis of the *TAP1* gene exon 3 G729A locus showed Sutai pigs deviated from the Hardy-Weinberg equilibrium. Sutai pigs have been specially bred to be resistant to disease, and the observed deviation from the Hardy-Weinberg equilibrium indicates that the general disease resistance had affected the equilibrium. In another study, Chen *et al.* found that the *TAP1* gene exon 3 G729A mutation of pigs had no significant impact on backfat thickness and growth traits (average daily gain, 0–100 kg) [25]. This further indicated that it was necessary to regard the G729A locus as an important molecular marker in piglets resistant to the *E. coli* F18 infection in Sutai pig breed.

## 4. Experimental Section

### 4.1. Experimental Materials and Sample Collection

#### 4.1.1. *Escherichia coli* F18-Resistant and *E. coli* F18-Susceptible Sutai Pigs

Previously, our research group used Sutai pigs to establish the *E. coli* F18 disease-resistant and the *E. coli* F18-susceptible resource populations that were subsequently studied by gene chips and microarray analysis [7,11]. In this experiment, using the V-secretion system and the receptor binding assay, further analysis and verification of *E.* *coli* F18 resistance and susceptibility were performed for these resource groups with the methods described in our published paper [11,12].

The samples in this study included eight *E.* *coli* F18-resistant piglets and eight *E.* *coli* F18-susceptible piglets that were obtained from the aforementioned verified *E. coli* F18-resistant and susceptible individuals. Piglets selected from the *E. coli*-resistant and susceptible resource groups and raised in the same environment were sacrificed postweaning (35 days). Fifteen centimeters of the duodenum and jejunum were obtained for the isolation and preparation of intestinal epithelial cells. Tissue samples were also taken from 11 organs, including the heart, liver, spleen, lung, kidney, stomach, muscle, thymus, lymph nodes, duodenum, and jejunum, and were stored in liquid nitrogen on-site and then transferred to a −70 °C freezer. 

#### 4.1.2. Other Experimental Samples

Based on genotyping results, twenty-four 35-day-old Sutai piglets (eight piglets per genotype) were selected and sacrificed, 11 tissue samples were collected from each of the piglets, snap frozen in liquid nitrogen, and stored at −70 °C. All the experiments were conducted in the Animal Hospital of Yangzhou University (China) according to the regulations for the Administration of Affairs Concerning Experimental Animals (Ministry of Science and Technology, China; revised in June 2012) and approved for experimental animal use (permit No. SYXK (Su) 2012-0029).

All the Sutai pigs were from Suzhou Sutai pig breeding center (Suzhou, China). Approximately 1.0 g of ear tissue was collected from each pig above and placed in 1.5 mL Eppendorf tubes and stored on ice before assay. Genomic DNA samples were extracted from ear notches according to a modified phenol/chloroform method [26].

### 4.2. Primer Design

Primers for PCR and real-time PCR for the *TAP1* gene were designed on the basis of the gene sequence obtained from the GenBank database (accession number: BX323833) [25]. All the primers were synthesized by Shanghai Invitrogen Biotechnology (Shanghai, China). *GAPDH* (*Glyceraldehyde-3-phosphate dehydrogenase*) was used as an internal control to normalize all of the threshold cycle (*C*_t_) values of other tissue products. Detailed primer information is listed in Table 4.

**Table 4:** Primer sequences and related information.

Gene	Method	Sequence (5'–3')	Annealing Temperature	Length
*TAP1*	PCR-RFLP	GAAATGTGGATAAGAGCA	63 °C	767 bp
		AAACAGACGGATAATGAAAGAGG		
*TAP1*	Real-time PCR	CCACTGCTTTTCCTTCTGCCT	60 °C	109 bp
		ACAGAACCTCAATGGCCACCT		
*GAPDH*	Real-time PCR	ACATCATCCCTGCTTCTACTGG	60 °C	187 bp
		CTCGGACGCCTGCTTCAC		

### 4.3. PCR-RFLP Analysis

The reaction mixture for the analysis included 20 μL of PCR product containing 100 ng of template DNA, 5 pmol of each primer, 10 μL of PCR Master Mix (Tiangen Biotech, Beijing, China), and sterilized distilled water to make up the final volume of 20 µL. The PCR protocol was as follows: 95 °C for 5 min; followed by 30 cycles of 94 °C for 40 s, 60 °C for 40 s, and 72 °C for 45 s; and an extension step at 72 °C for 10 min. The PCR products were verified by electrophoresis in 1% agarose gel stained with ethidium bromide. Then, 10 µL of each PCR product was digested by incubation with 2 U of *Mbo*I (Sangon, Shanghai, China) restriction enzyme at 37 °C overnight. The digested fragments were electrophoresed in 10% polyacrylamide gels in 1× TBE (Electrophoresis buffer: Tris base 54 g/Boric acid 27.5 g/EDTA 3.72 g, added ddH_2_O to 5 L; pH = 8.0) at a constant voltage of 120 V, stained by silver, and visualized under ultraviolet light.

According to the results of the PCR-RFLP analysis, PCR products of different homozygotes were sequenced by Biological Engineering (Shanghai, China) in an ABI PRISM 377 automatic DNA sequencer (Applied Biosystems, Foster City, CA, USA).

### 4.4. Total RNA Extraction and Real-Time PCR

Total RNA was extracted from various swine tissues (50–100 mg) using Trizol reagent (TaKaRa Biotechnology, Dalian, China) according to the manufacturer’s instructions. Precipitated RNA was dissolved in 20 μL of RNase-free H_2_O and stored at −70 °C. Qualitative and quantitative assessment of RNA was carried out by agarose gel electrophoresis and NanoDrop 1000 Spectrophotometer (NanoDrop Technologies, Inc., Wilmington, DE, USA), respectively.

The 10 µL reaction mixture for cDNA synthesis contained 2 µL of 5× PrimerScript buffer, 0.5 µL of PrimerScript RT Enzyme Mix I, 0.5 µL of Oligo dT, 0.5 µL of random hexamers, 500 ng of total RNA, and RNase-free H_2_O to make up the final volume of 10 µL. The reaction was carried out at 37 °C for 15 min and at 85 °C for 5 s.

Real-time PCR amplification was performed in a 20 µL reaction mixture containing 1 µL of cDNA (100–500 ng), 0.4 µL of each forward and reverse primer (10 μM each), 0.4 µL of 50× ROX Reference Dye II, 10 µL 2× SYBR Green Real-time PCR Master Mix, and 7.8 µL double-distilled H_2_O. The PCR protocol was as follows: 95 °C for 15 s, followed by 40 cycles of 95 °C for 5 s, and 62 °C for 34 s. The dissociation curve was analyzed after amplification. A *T_m_* peak at (85 ± 0.8) °C on the dissociation curve was used to determine the specificity of the PCR amplification. The *T_m_* value for each sample was the average of the real-time PCR data for triplicate samples.

### 4.5. Data Processing and Analysis

The frequencies of gene and genotype were calculated according to Hardy-Weinberg equilibrium principle: *p* = *P* + *H*/2, *q* = *Q* + *H*/2, *x*^2^ = Σ*d*^2^/*e*; *d* = *e* − *o*, which is the difference between predicted value and detected value; *p* and *q* represent allele frequency at certain position.

The average expression level of the *TAP1* gene in the *E. coli* F18 susceptible individuals’ muscle was defined as 1.0 between the *E. coli* F18 resistant and susceptible groups, and the average expression level of the *TAP1* gene in the AA genotype individuals’ muscle was defined as 1.0 among 3 genotype groups, so that the relative expression levels of this gene in other tissues could be quantified. The 2^−ΔΔ*C*t^ method was used for processing the relative quantification results [27].

The statistical analyses were carried out using the SPSS 15.0 software (SPSS, Chicago, IL, USA) and shown as *x* ± SD. The *t-*test was carried out to determine the significance of differences in mRNA relative expression between the resistant and susceptible groups, and the general linear model (GLM) was carried out to determine the significance of differences in mRNA relative expression among three different genotype individuals.

## 5. Conclusions

According to the results of our research, compared with susceptible individuals, the high expression of the *TAP1* gene among resistant piglets might benefit the individuals to prevent *E. Coli* F18 infection. The mutation on the *TAP1* gene G729A would affect the expression of the *TAP1* gene, and the GG genotype individuals have higher expression levels. Therefore, we could speculate that GG genotype individuals might have better ability of *E. Coli* F18 resistance, and the *TAP1* gene G729A mutation should be regard as one genetic marker for further study in pig disease resistance breeding.
